# *De Novo* Mutations in Ataxin-2 Gene and ALS Risk

**DOI:** 10.1371/journal.pone.0070560

**Published:** 2013-08-06

**Authors:** José Miguel Laffita-Mesa, Jorge Michel Rodríguez Pupo, Raciel Moreno Sera, Yaimee Vázquez Mojena, Vivian Kourí, Leonides Laguna-Salvia, Michael Martínez-Godales, José A. Valdevila Figueira, Peter O. Bauer, Roberto Rodríguez-Labrada, Yanetza González Zaldívar, Martin Paucar, Per Svenningsson, Luís Velázquez Pérez

**Affiliations:** 1 Center for the Research and Rehabilitation of Hereditary Ataxias, Holguín, Cuba; 2 Branch of Biomedicine of the Cuban Academy of Sciences -ACC-, Havana, Cuba; 3 Clinical and Surgical Hospital Lucía Íñiquez Landín, Holguín, Cuba; 4 Laboratory of Virology of Department of ITS, Institute of Tropical Medicine Pedro Kourí, Havana, Cuba; 5 Department of Clinical Neuroscience, Center for Molecular Medicine, Karolinska Institute, Sweden; 6 Department of Neuroscience, Mayo Clinic, Jacksonville, Florida, United States of America; 7 Policlinic and Medical Faculty, Tacajó, Báguanos, Holguín, Cuba; Pasteur Institute of Lille, France

## Abstract

Pathogenic CAG repeat expansion in the ataxin-2 gene (*ATXN2*) is the genetic cause of spinocerebellar ataxia type 2 (SCA2). Recently, it has been associated with Parkinsonism and increased genetic risk for amyotrophic lateral sclerosis (ALS). Here we report the association of *de novo* mutations in *ATXN2* with autosomal dominant ALS. These findings support our previous conjectures based on population studies on the role of large normal *ATXN2* alleles as the source for new mutations being involved in neurodegenerative pathologies associated with CAG expansions. The *de novo* mutations expanded from ALS/SCA2 non-risk alleles as proven by meta-analysis method. The ALS risk was associated with SCA2 alleles as well as with intermediate CAG lengths in the *ATXN2*. Higher risk for ALS was associated with pathogenic CAG repeat as revealed by meta-analysis.

## Introduction

Amyotrophic lateral sclerosis (ALS), also known as Lou Gehrig’s disease, is a devastating adult-onset neurodegenerative disease with no cure and is fatal within 2 to 5 years after the disease onset. Phenotypically, it is characterized by progressive motor neuron loss resulting in muscle weakness, wasting, fasciculations, spasticity, and hyperreflexia. The vast majority of cases (∼90%) have no family history, while about 10% of patients have a genetic locus as causal entity for ALS. So far, mutations in *SOD1*, encoding for Cu/Zn superoxide dismutase, have been identified in 20% of familial ALS cases. Other frequent disease-causing genes include *C9ORF72*, TAR DNA-binding protein 43 (*TARDBP*), and fused in sarcoma/translocated in liposarcoma protein (*FUS/TLS*) [1]. Recently, it has been shown that large intermediate CAG repeat expansions in the ataxin-2 gene (*ATXN2*) contribute to almost 5% of the sporadic or familial ALS cases [2]. Intermediate CAG repeats are now recognized as ALS13 associated locus (MIM183090). Based on several studies, it appears that CAG expansion in *ATXN2* might account for more familial ALS cases than *SOD1* mutations with an overall incidence of 2% [2]–[5]. SCA2 is another neurodegenerative disorder with no available cure exhibiting progressive cerebellar syndrome characterized by ataxic gait, cerebellar dysarthria, dysmetria, and dysdiadochokinesia.

Normal CAG length in *ATXN2* gene ranges from 13–31 repeats, with 22 CAG being most common. Alleles with ≥26 CAG are considered as large alleles [6]. While inconsistencies exist in the literature regarding the CAG range and nomenclature of the intermediate or indeterminate alleles, those with ≥27–33 CAG are associated with increased and specific risk for ALS [2], [3], [7].

Only few cases with 32–34 CAG expansions have been reported so far in SCA2 [8]–[11]. Of note, none of the individuals with intermediate allele displayed typical clinical SCA2 picture. Therefore, starting point for SCA2 mutant range has been widely considered ≥34 CAG, while 37–75 CAG repeats are full penetrant [12] and massive expansions has been observed with severe disease starting in infants [13], [14].

First report relating ALS with intermediate alleles considered 27 repeats as the threshold [2] while subsequently it has been refined to >30 CAG [3], [7]. However, in SCA2, these alleles are considered as large normal, which might be prone for intergenerational instability leading to full penetrant disease-causing alleles, but this instability has not been reported so far. Given the rarity of large and intermediate alleles (23–34 CAG), little is known about the origin of ALS-related alleles. Only one *de novo* mutation has been reported, but the data about the unstable behavior of the involved alleles were not revealed [5]. Investigation in these patients would be important to understand events leading to genetic instability and for the accurate presymptomatic and prenatal diagnostics as well as genetic counseling.

Recently, we have shown that the highest worldwide concentration of large normal alleles (≥23 CAG) underlies the highest worldwide prevalence and incidence rates of SCA2 in Cuba. Based on this observation we postulated that *ATXN2* intermediate alleles with 32–35 CAG may evolve from large ANs with 23–31 CAG, explaining familial or sporadic cases associated either with SCA2, ALS, and Parkinsonism [5]. In support of our postulates, we uncovered familial ALS cases resulting from large unstable alleles reaching ALS intermediate length and SCA2 pathogenic expansions at the *ATXN2* locus. In this study, we provide data on the global risk of intermediate *ATXN2* alleles for ALS.

## Results

### Clinical Phenotype of ALS with *de novo* CAG Expansions in Ataxin-2 Gene

**Figure 1:**
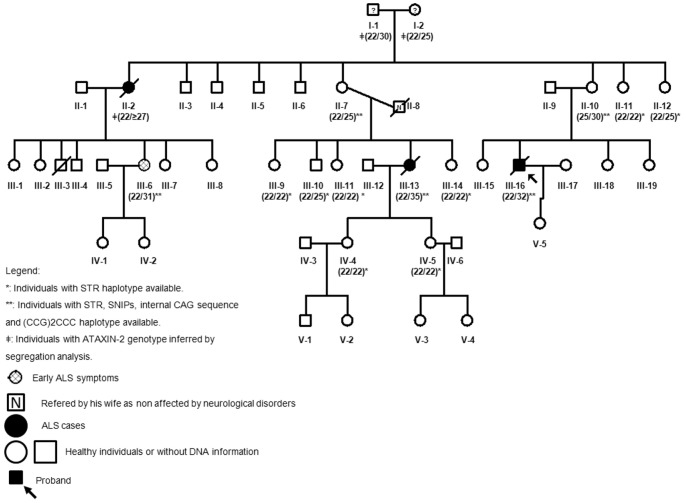
Pedigree of family in which novel sequence variants and three *de novo* mutations were identified.

We identified a pedigree with three ALS (two fully developed ALS: III-13 and III-16; and one in early stages of ALS: III-6) cases segregating with intermediate *ATXN2* CAG repeat which originated from unexpanded allele (Fig. 1). Average age at onset in this family was 54.25±1.26 years and the survival was 25±21.28 months. Ataxia, slow saccades or other symptoms which would indicate co-morbidity of ALS and SCA2 were not found in these patients. Clinical records of two cases showed neither ataxic signs nor cerebellar anomalies excluding SCA2 presenting initially as motor neuron disease. Additional clinical information of these cases is presented in Table 1 and described below.

**Table 1:** Phenotype and *ATXN2* genotype for ALS case series.

Case	Age	Clinical status	Gender	Ataxin-2 Genotype	D12S1333	D12S1672	D12S1332
I.1/I.2	–	Healthy	Female/Male	[22/25]	[251]	[289]	[190]
					NA	NA	NA
II.2	54	ALS*	Female	[22/≥27]	[239]	[289]	[198]
					NA	NA	NA
II.7	76	Healthy	Female	22/25	245	125	170
					251	289	190
II.10	72	Healthy	Female	22/25	239	125	170
					257	289	190
II.11	67	Tremor	Female	22/22	245	125	170
					245	289	198
II.12	63	Healthy	Female	22/25	231	125	170
					251	289	190
III.6	57	ALS	Female	22/31	231	129	170
					239	289	198
III.9	59	Healthy	Female	22/22	227	287	202
					245	125	170
III.10	57	Healthy	Male	22/25	245	125	170
					257	289	190
III.11	50	Healthy	Female	22/22	245	125	170
					257	283	196
III.13	54	ALS	Female	22/35	239	133	172
					239	289	202
III.14	38	Healthy	Female	22/22	245	125	170
					231	289	202
III.16	54	ALS	Male	22/32	231	129	170
					239	289	198
IV.4	38	Healthy	Female	22/22	239	133	172
					245	287	196
IV.5	29	Healthy	Female	22/22	239	133	172
					245	185	202

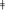
Affected by history and genotype inferred from daugther.

* De novo mutation causing disease. **EERC**: El Escorial Revised Classification.

**Figure 2:**
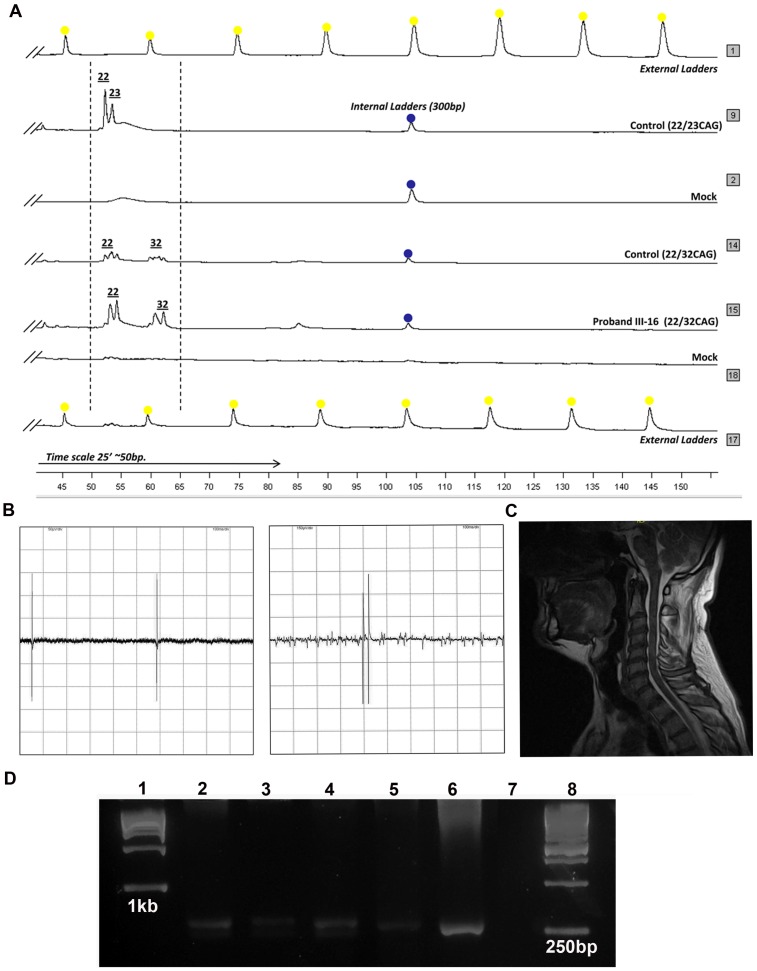
Genetics, EMG and MRI analysis. A) Electrophoresis of fluorescent fragment analysis of *ATXN2* CAG repeat. In each lane, the content is specified. B) Fasciculation patterns in proband’s tongue and in biceps brachii recorded by EMG. C) Midsagittal MRI image of proband, no cerebellar atrophy is evident. D) Representative 3% agarose electrophoresis of *C9ORF72* analysis in index case and parents (lanes 2, 3, 4). Lanes 1and 8: MW markers (Ready Load™ 1–12.216 Kb ladder and 250–3500 bp ladder in multiples of 250 bp (Invitrogen), respectively; Lane 7: mock; lanes 2, 3, 4: case III-16, and both parents II-9 and II-10, respectively. Note that each DNA showed two defined bands despite PCR products with 7-deaza-2-deoxy GTP stain poorly with ethidium bromide. These 3 samples were heterozygous with bands higher than 250 bp but bellow 350 bp (hex repeat ∼11units) using Renton et al. primers anchoring 280 bp from hex-repeat. Lanes 5, 6 are unrelated ALS cases from the Cuban population.

The proband (III-16), appeared as a sporadic ALS case when assisted by consultants. This patient from pedigree A1 first experienced weakness and pain in the right heel at age of 53 years, progressing to paralysis of the leg, the left leg was also compromised within another month. MRI was indicated and misdiagnosis of lumbar radiculopathy was set. Treatment with non-steroidal anti-inflammatory drugs and Vitamin-B12 did not lead to an improvement. Four months later, upper right limb was started to be affected with wasting and weakness and within a month, it progressed to the left side. In all four extremities, hyperreflexia (+4) and moderate spasticity was present. Marked distal hypotrophy at first dorsal interosseum in both upper and lower limbs was observed with severe deterioration of first interosseum as well as in thenar and hypothenar eminences. Right hand adopted simian-like shape, requiring right-to-left hand change. About 6 months later, atrophy of arms, forearms, deltoid, and pectoralis muscles, accentuated pyramidalism featured by hyperreflexia, bilateral Hoffman and Troemmer signs predominating on the right side and Babinski reflex were evident. Sensation and coordination were normal. Both heels showed non-exhausting and exhausting clonus. Bulbar involvement was confirmed with remarked atrophy of the tongue edges, and tongue and palate fasciculations. Somatosensory evoked potentials of upper and lower limbs were normal. Electromyography (EMG) in muscles in all extremities (m. biceps brachii, gastrocnemius, tibialis, rectus femoris, pectoralis, spinalis) and the tongue showed diffused signs, widespread fasciculations, fibrillations and sharp-waves. At rest, there were spontaneous discharges of motor unit action potentials in interosseal muscles. On contrast, dropout of the number of motor units with increased firing rate was evident. In summary, EMG report was indicative of neurogenic changes with signs of active denervation and renervation, spontaneous fasciculations (Fig. 2B) and fibrillations in all four limbs and bulbar segments, reflecting acute and progressive denervation of the second motor neuron of the brain stem and the anterior horn of spinal cord and a definite diagnosis of a classical ALS was concluded. Magnetic resonance imaging showed generalized cortical atrophy (not shown). No cerebellar damage was evident on a recovered sagittal MRI image.

Weakness in all the extremities progressed, and the patient subsequently developed dysarthria and dysphagia. Approximately 7 months later, the patient started to experience gait and later stance abnormalities and became quadriplegic, anarthric and aphagic. Neurological examination showed that patient was alert and oriented until his death. At this clinical stage he showed vasomotor disturbance congruent with Sudeck’s atrophy. The entire clinical picture progressed several months until death caused by respiratory failure at age of 55 years. *ATXN2* CAG repeats length expansion was 22 and 32 CAG repeats (Fig. 2A, lane 14, Table 1) with the longer allele belonging in the intermediate range associated with ALS.

The sister of proband’s mother (II-2) lived between years 1933–1991 and started to experience similar symptoms at age of 54 years. The disease progressed to death within 4 years (at age of 58 years). According to the clinical records her first symptom was hoarseness progressing throughout∼15 years with pain (at age of 54 years) in the right heel and weakness in arms and legs. As she deteriorated, the course of her clinical picture occurred without dementia, cognitive or social dysfunction. A diagnosis of lumbar radiculopathy was proposed based on tomography performed in 1989. She subsequently developed dysarthria and dysphagia, was confined to wheelchair, and died due to respiratory failure. Although her genetic material was not available, a mutation near or beyond 31 CAG in *ATXN2* could be suspected, as her daughter’s (III-6) genetic status of *ATXN2* is 22/31 CAG. Importantly, III-6 referred that, similarly to her mother, she has been suffering from progressive weakness and pain in lower limbs in last 7 months. Additionally, she complained about frequent cramps in the right arm and leg. At neurological examination, no signs of ataxia signs were found. She had motor neuron signs in all extremities and provoked fasciculations were evident in arms. Muscular strength in limbs was: superior limb was 4/5 distal, 4/5 proximal bilateral while in right inferior limb 4/5, 4/5 proximal bilateral and in left 4/5 distal. This clinical information is in agreement with early symptoms involving bombardment of motor neurons progressing toward typical ALS. In addition, her status highlights the possibility that the same genetic variants in *ATXN2* causing ALS in her mother might be the responsible for her clinical signs indicative for ALS. EMG was not performed in this patient due to her disapproval of this examination. While the definite ALS diagnosis in II-2 cannot be rule out, and was confounded in her time with lumbar radiculopathy, it seem clear that she presented a progressive neurodegenerative disorder involving motor neurons, and for her interviewed relatives this presentation resembled the picture of her first order nephews, our proband and III-13 (see below), both with a motor-neuron disease and where a hereditary pattern can be applied.

The examination of the patient III-13 (cousin of III-16) revealed weakness in the right leg and dysarthria lasting approximately 4 months. Similarly to the previous cases, the symptoms occurred at age of 54 years and led to death within 6 months. For about 3 months, the patient experienced muscle twitching in upper limbs and in m. gastrocnemius, as well as dysarthria, dysphagia and mild tongue atrophy. Neurological examination revealed spontaneous and provoked fasciculations in upper limbs, m. gastrocnemius and spinal muscles. Muscular strength in right upper limbs was 3/5 distal, 4/5 proximal bilateral while in right lower limb 3/5, 4/5 proximal bilateral and in left 4/5 distal. Both spatia interossea displayed hypotrophy with more profound picture on the right side and with marked deterioration in the first interosseum. Hyperreflexia (+3) and exhausting clonus in both heels was also observed. Babinski signs were discrete on the right side. Cutaneous sensibility was normal. On neuropsychological grounds cognitive and social function were preserved. Somatosensorial evoked potentials of upper and lower limbs were normal and MRI only showed generalized cortical atrophy and similarly to III-16 EMG of both upper and lower limb muscles (m. tibialis anterior, vastus medialis, gastrocnemius, first dorsal interosseous and biceps brachii) suggested denervation of second motor neuron compatible with ALS. On muscle contraction, decrease of recruitment pattern was evident, and in addition to motor neuron anomalies shown in III-16, a remarkably clear neurogenic EMG record was obtained in tongue all compatible with definite ALS. Genotype analysis revealed 22/35 CAG repeat size in *ATXN2* (Table 1), which is in the pathogenic range for SCA2. Since the first consultation, the clinical picture worsened progressively until quadriplegia in the last 3 months and the patient’s death at age of 55 years due to respiratory failure.

All these cases showed intermediate *ATXN2*≥31 CAG, which are scattered in 80 chromosomes from the general population (frequency 0.025, and see Supplementary Figure S2 in reference 6) but overrepresented only in the SCA2/New Mutation deriving sample [6]. In that study an isolated allele with 35 CAG was found with no connections to SCA2 founder families [6], and as far as we know, the carrier has not developed neurological signs. This patient is now 37 years old, 18 years below the average onset age of ALS individuals presented in this study.

### Segregation Analysis of Intermediate-length *De Novo* Mutated Alleles

**Figure 3:**
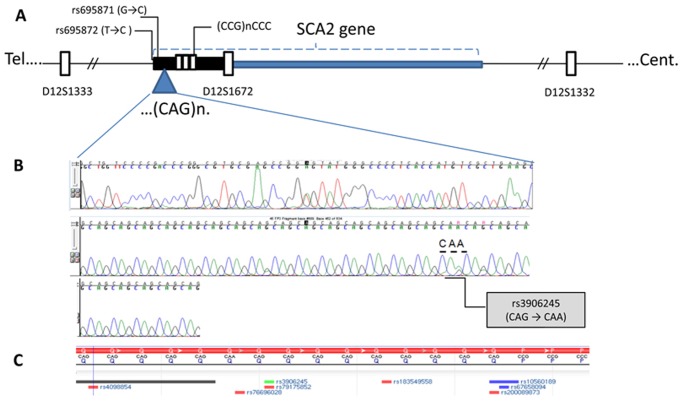
Genetic markers for *ATXN2* haplotyping and gene sequencing. A) *ATXN2* gene schematic maps and microsatellite, D12S1333 (telomeric at 200 kb from *ATXN2*), D12S1672 (intragenic at exon 1) and D12S1332 (centromeric at 350 kb from *ATXN2*) and SNIPs markers, rs695871 (at 177 bp upstream CAG expansion), rs695872 (at 106 bp upstream CAG expansion) and rs390624 (within the expanded CAG) used for haplotyping cases involved in *de novo* mutations, the polymorphic (CCG)nCCC/poly-proline adjacent to the CAG expansion is also indicated. B) Sequencing for case II-10, mother of the proband III-16 (25 CAG with only one CAA interruption). C) Relative position for other SNPs situated either within or near the expanded CAG.

DNA for genetic analysis was available from sib-ship pairs and revealed that at least three *de novo* mutations occurred. They have been proven in two patients (III-13 and III-16) and in one it was inferred (II-2) (Fig. 1). Female II-2 was the first person in the family with ALS symptoms and she was the first daughter of I-1 and I-2 deceased at age of 87 and 79 years, respectively, with no neurological disorders referred by their children (Fig. 1). They had eight other children (Fig. 1) with following *ATXN2* CAG repeat sizes: 22/25 (II-7), 25/30 (II-10), 22/22 (II-11) and 22/25 (II-12). The genetic material from II-2, II-3, II-4 and II-5 was not available. Neurological symptoms were observed only in one sibling (II-11) while the others appeared healthy despite advanced ages at examination (II-7, 63 years; II-10, 72 years; II-11, 67 years; II-12, 76 years). Siblings II-3, II-4 and II-5 were not examined as they live abroad. According to the family members, they are all healthy. Individual II-11 showed resting and kinetic tremor unrelated with ataxin-2 (normal genotype of 22/22 CAG) (Fig. 1) and different haplotypes in each allele (Table 2). According to the segregation analysis of this pedigree, the most probable genotype in the parents was 22/25 and 22/30. As an evidence for parental genotype serves the fact that the alleles with 25 and 30 CAG had different STR haplotype in the healthy 72-years old daughter (II-10) who is also the mother of the proband III-16 (Table 2). Moreover, 22/22 CAG and 22/25 CAG combinations were found in the other children (e.g. 22/25 in II-7, 22/22 in II-11, and 22/25 in II-12), suggesting the presence of a fourth allele in parents with 22 CAG repeats (for haplotypes see also Table 2 and Fig. 3A).

**Table 2:** Table. 2. Microsatellite haplotypes.

No.	*G*enotype	Gender (Birth)	Clinical status	Age at onset/death	Survival (mo)	Site of onset, EERC
II-2	22/≥30*	Female (1933)	Affected 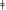	54/58	48 (deceased)	Spinal right (lower limb), ALS possible
III-6	22/31	Female (1955)	Early symptoms	56, Alive	–	–
III-16	22/32*	Male (1956)	Affected	53/55	21 (deceased)	Spinal right (lower limb), ALS definite
III-13	22/35*	Female (1957)	Affected	54/55	6 (deceased)	Spinal right (lower limb), ALS definite

NA: Not available, Inferred haplotype are bracketed,

* sick by history.

**Figure 4:**
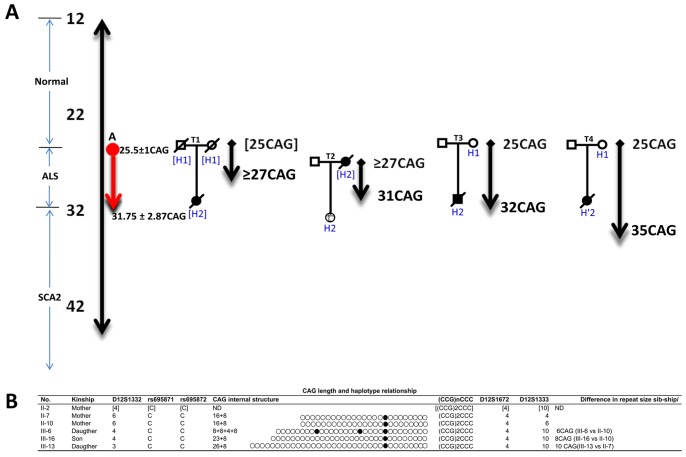
CAG repeat instabilities and *de novo ATXN2* mutations associated with ALS in relationship with the haplotype. A) Schematic presentation of the CAG instabilities in four transmissions (T1–T4) of SCA2 (drawn to scale). Haplotypes are indicated as H1, H2, H′1and H′2 (see explanation in the text). Red (A), represents the average for CAG instabilities in the four transmissions. All transmissions reach either ALS (27–33 CAG) or SCA2 (≥34 CAG) thresholds. B) Scheme representing the haplotype and CAG(CCG)2CCC sequence for the individuals involved in the unstable CAG transmissions. Data between brackets is inferred from the segregation analysis. D12S1332, 3: 202 bp, 4: 198 bp, 6: 190 bp, D12S1672, 4: 289 bp, D12S1333, 4: 257 bp, 6: 251 bp, 10: 239 bp.

One of the longer alleles (25 or 30 CAG) would segregate in II-2 as a *de novo* ≥27 CAG ALS mutation which was further transmitted to III-6 (22/31 CAG) and might be responsible for the early signs of ALS (transmissions T1 and T2 in Fig. 4). The allele with 30 CAG could be suggested as the possible source for *de novo* ALS cases with *ATXN2* CAG expansions. However, segregation analysis for the transmission T4 (Fig. 4A) conformed by II-7 (22/25 CAG) and III-13 (22/35 CAG) revealed that instead of the 30 CAG allele, the one with 25 CAG in II-7 (age 76 years) underwent a pathogenic expansion by 10 CAG and contributed to ALS onset at age of 54 years. This *de novo* mutated allele in III-13 crossed the threshold for ALS and reached the minimal repeat length for SCA2 (34 CAG) (Fig. 4A). The origin of these expansions from the 25 CAG allele with one CAA interruption was also confirmed in the unstable transmission T3, sib-ship pair of II-10 (25/30 CAG)/III-16 (22/32 CAG) (Fig. 3B, Fig. 4A,B) expanded by 8 repeats and was found associated with classical ALS phenotype described above. The contribution of proband’s father to *ATXN2* mutagenesis is not likely as he was a carrier of 22/22 CAG alleles. One of these alleles was identified in proband with 8+4+8 CAG/CAA pattern. Despite the absence of genetic data in mother of III-6, we could identify the increase in CAG length by 6 repeats as compared with CAG in the aunts (II-7 and II-10) (Fig. 4B). Normal CAG expansions were found in two of her sisters (III-2 and III-7) with22/22 CAG genotype. In summary, the average for intergenerational instability in all described transmissions was 6.25±2.87 gained CAG units in alleles sized 25.5±1 CAG, resulting in *de novo* mutated alleles with ∼32CAG (transmission A in Fig. 4A) which is a greater CAG repeat than the threshold for ALS risk ≥30 CAG [3], [7].

### Haplotype of Ataxin-2 Intermediate-length Polyglutamine Expansions

To clarify the mechanism of *de novo* CAG mutations, we performed SNIPs and STR haplotyping. The analysed markers span ∼550 kb and are lined up on chromosome 12q24.1 as follows: centromere-D12S1332-D12S1672-(CCG)nCCC-CAG-rs695871-rs695872-D12S1332-telomere (Fig. 3A). The mothers of patients III-13 and III-16 (II-7 and II-10) shared the truncated haplotype 6-4 (289 bp and 257 bp) for markers D12S1672 and D12S1332 embedding the 350 kb region including the SCA2 gene (Fig. 3A,B). For the D12S1333 telomeric marker at 20 kb, variation of 3 CA/GT dinucleotides was found (allele 4, 257 bp and allele 6, 251 bp), yielding closely related haplotype variants H1 (6-4-4) for II-7 and H′1 (6-4-6) for II-10.

As for the intergenerational transmissions, STR haplotype analysis revealed that all CAG instabilities involving parental haplotypes H1 or H′1 lead to significant changes in offsprings with the resulting haplotype H2: 4-4-10 in III-6, III-16 and H′2: 3-4-10 in III-13 (Fig. 4A,B). Further DNA haplotyping in III-6 showed that H2 variant was shared with III-16 and II-2.

The haplotypes in generation III are distinct from the original parental haplotypes regarding the microsatellites D12S1333 and D12S1332 but not the intragenic marker D12S1672. The more significant changes of STR were evident in the vicinity of SCA2 locus, for example D12S1333 decreased 9 and 6 CA/GT and D12S1332 increased 6 and 4 CA/GT dinucleotides in III-13, III-6 and III-16, respectively, while no changes were seen in D12S1672 marker (Figure 4A,B).

As for the intragenic SNIPs and the polymorphic CCG/CCC tract, we found C-C and (CCG)_2_CCC variants for SNIPs rs695871 and rs695872, respectively, as the haplotype co-segregating with the intermediate and the normal (25 CAG) alleles (Fig. 4B, Table 2).

These results showed that the intragenic markers (SNIPs and D12S1672) are conserved but the flanking markers (D12S1332, D12S1333) are variable in the transmitted alleles, i.e.: 6-C-C-4-Intermediate CAG-(CCG_2_CCC)-6/4 and 3/4-C-C-4-Intermediate CAG-(CCG_2_CCC)-10, respectively.

### Mechanism of De Novo Mutation: the Role of CAA Interruption Pattern

Next, we analyzed CAA interruptions of the CAG tract by DNA sequencing. The interruption patterns were as follows: 16+8 (II-7 and II-10), 23+8 (III-16), 26+8 (III-13), and 8+8+4+8 (III-6) (Fig. 4B). This analysis suggested sequential lengthening of the 5′ repeat tract in the alleles with the concurrent absence of the most proximal CAA interruption. Lack of this interruption enhances the enlargement of CAG tract, but no instability was found in the (CCG)_2_CCC triplets flanking the CAG repeat (Fig. 4B).

### Ataxin-2 Intermediate Alleles and ALS Risk

**Figure 5:**
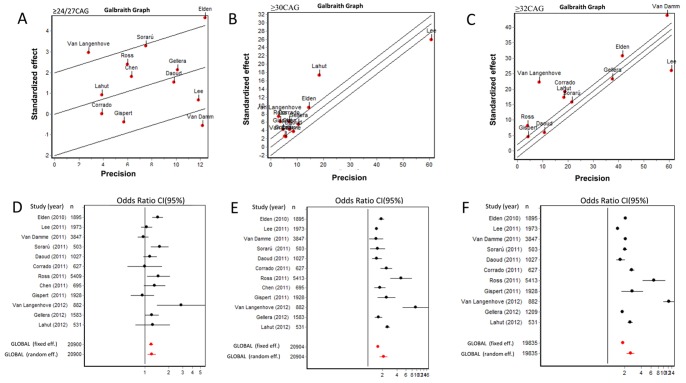
Meta-analyses for large and intermediate CAG lengths in *ATXN2*. Galbraith (A–C) and forest plots (D–F) for different *ATXN2* CAG length across populations. A) and D) ≥24/27 CAG. B) and E) ≥30 CAG. C) and F) ≥32 CAG.

**Figure 6:**
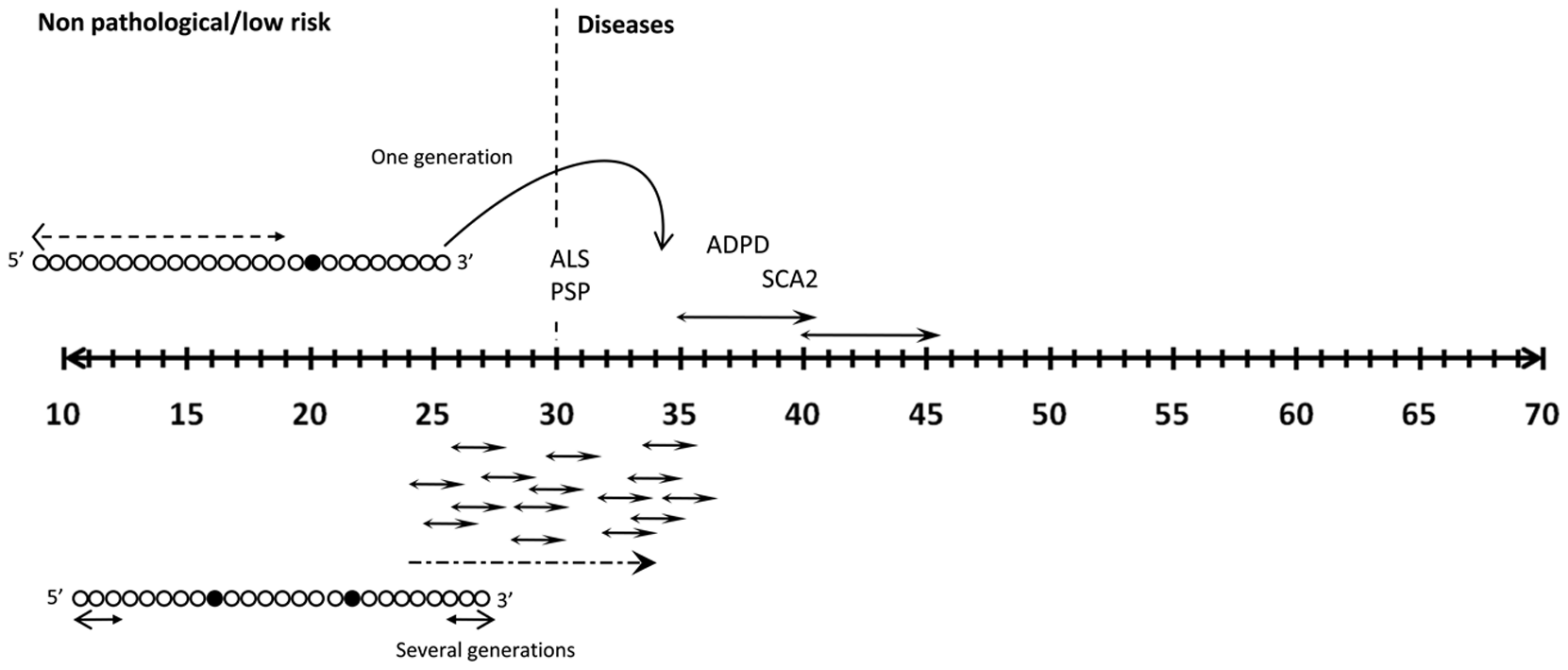
General mechanisms for *ATXN2* gene *de novo* mutagenesis in the population. Two models can be proposed for explanation of *de novo* CAG expansions in *ATXN2*. Both involve loss of the CAA interruption in large alleles resulting in a minimal length of pure repeat within the CAG expansion. CAA interruptions break the CAG tract in discrete repeat arrays protecting it from instability [17]. According to this study, the minimal length of the internal pure repeat leading to *de novo* mutations is 8 CAG.

Genetic overlap between SCA2 and ALS has been reported [5] without co-segregation of mutations in the most common ALS-related genes (*C9ORF72*, *TDP43*, *FUS*,*UBQLN2*,*ANG*,*OPTN*, *SPG11*, *PLEKHG5*, *VAPB*) in ALS cohorts with *ATXN2* intermediate-length (Table S1). We also found no segregation of *C9ORF72* hexanucleotide repeat expansion in our cases. Moreover, *de novo* mutations in our pedigree originated from the 25 CAG allele, which we consider as “large normal” [6] while other authors as intermediate ALS-risk alleles [2].Therefore we investigated whether this allele, which is overrepresented in our population [6], could be considered as a risk factor for ALS. Meanwhile, we aimed to address the refinement of currently accepted threshold of intermediate *ATXN2* alleles as risk factors for ALS. In the first step of the meta-analysis we used 24 or 27 CAG as threshold for ALS risk (Table 3). The combined risk was heterogeneous among 12 studies with χ^2^ = 29.97, df = 11, P = 0.0016. At this starting point we detected a higher prevalence of intermediate alleles in the ALS cases as compared with controls (OR = 1.23 95% CI 1.09–1.39). This modest value of association is also supported by the appearance of healthy controls with intermediate alleles and low risk for this range in some populations [15]. In the population study in Belgium and Netherlands, the contribution of the 30 CAG alleles to the ALS risk has been shown, although the threshold at 27 CAG was confirmed [5] (Table 3). To clarify this, we performed meta-analysis with CAG range between 24 and 27 repeats, and the heterogeneity of the risk alleles dropped (χ^2^ = 16.95, df = 11, P = 0.11) (data not shown) with no significant risk of the alleles in this range (OR = 1.02; 95% CI 0.91–1.16). These results point to >27 triplet repeats length as one of the sources for heterogeneity seen in the first meta-analysis with threshold between 24 and 27 CAG. Furthermore, the data confirmed that the *de novo* mutations reported in this study originated from alleles with low or no risk for ALS with a propensity to cross the pathogenic range in further generations (Fig. 5A, Table 3 and Fig. 6).

**Table 3:** Meta-analysis for case-controls series for ATXIN-2 polyQ intermediate length and ALS.

Study, Population	ALS cases/Controls (ratio)	ATXN2 polyQcutoff, P-value*	PolyQ>24/27OR (95%CI)	24≥PolyQ≤27OR (95%CI)	PolyQ≥30OR (95%CI)	PolyQ≥32OR (95%CI)
Elden et al., 2010, North America [2].	915/980 (0.93)	≥27, 3.6×10^−5^	1.45 (1.24–1.70)	1.24 (0.99–1.55)	1.91 (1.67–2.19)	2.08 (1.99–2.18)
Lee et al., 2011, Northern Europe [3].	1294/679 (1.91)	>30, 6.2×10^−3^	1.06 (0.90–1.25)	0.94 (0.75–1.16)	1.53 (1.48–1.58)	1.53 (1.48–1.58)
Van Damme et al., 2011, Belgium and theNetherlands [5].	1845/2002 (0.92)	≥32, 3.6×10^−2^	0.96 (0.81–1.12)	0.91 (0.76–1.08)	1.49 (1.07–2.08)	2.09 (2.02–2.16)
Sorarú et al., 2011, Italy [28].	247/256 (0.96)	≥24, 2.6×10^−2^	1.54 (1.19–2.00)	1.47 (1.05–2.08)	1.60 (1.12–2.30)	2.06 (1.88–2.25)
Daoud et al., 2011, French andFrench Canadian [19].	556/471 (1.18)	≥29, 2.4×10^–4^	1.17 (0.96–1.42)	1.02 (0.79–1.33)	1.53 (1.22–1.93)	1.71 (1.43–2.04)
Corrado et al., 2011, Italy [4].	232/395 (0.59)	>30, 8.9×10^−4^	1.00 (0.61–1.65)	0.42 (0.15–1.19)	2.41 (1.82–3.19)	2.73 (2.46–3.03)
Ross et al., 2011, North America [17].	536/4877 (0.11)	>30, 1×10^−3^	1.49 (1.07–2.06)	1.19 (0.82–1.74)	4.81 (2.89–8.01)	6.8 (4.25–10.87)
Chen et al., 2011, Chinese [18].	345/350 (0.99)	≥32, 4×10^−2^	1.33 (0.98–1.81)	1.08 (0.67–1.74)	1.78 (1.35–2.34)	–
Gispert et al., 2012, Europe [7].	559/1369 (0.41)	≥30, 4×10^−3^	0.93 (0.66–1.32)	0.75 (0.50–1.13)	2.43 (1.61–3.67)	2.77 (1.78–4.32)
Van Langenhove et al., 2012,Flanders-Belgian [16].	72/810 (0.09)	≥30, 1.2×10^−2^	2.86 (1.43–5.73)	1.79 (0.70–4.57)	9.54 (5.19–17.55)	12.41 (9.93–15.51)
Gellera et al., 2012, Italy [20].	658/551 (1.19)	>30, 1.4×10^−3^	1.23 (1.02–1.50)	0.92 (0.68–1.24)	1.66 (1.38–2.00)	1.85 (1.75–1.95)
Lahut et al., 2012, Turkish [15].	212/319 (0.66)	>30, 2.6×10^−2^	1.26 (0.76–2.08)	0.83 (0.37–1.86)	2.53 (2.28–2.82)	2.53 (2.27–2.81)

* As reported by authors in the original data.

When the meta-analysis was performed using repeat length threshold of ≥30 CAG, the ALS risk was estimated more accurately (Fig. 5B). We detected specific risks estimates in all populations (Table 3) and a higher global risk (OR = 2.16; 95% CI 1.76–2.65, χ^2^ = 187.88, P = 0.000) representing a two-fold excess of intermediate alleles enriching ALS cases versus healthy control population. Excluding the most precise studies [2], [3], [15] (Figure 5B) and those with the most extreme odds ratios [16], [17] (Table 3) resulted in homogenous relative risk >1 (P = 0.12), which confidently input positive combined risk of 1.77; 95% CI 1.55–2.03 for intermediate allele in ALS patients versus controls. Although the risk for ALS was associated with the ≥30C AG alleles, it was higher in carriers of ≥32 CAG repeats (Fig. 5C,F) in all populations except Chinese [18] with no alleles with ≥32 CAG. The combined risk was “significantly heterogeneous” (2.62; 95% CI 2.23–3.09, χ^2^ = 633.97, df = 10, P = 0.0000) and eight cases-control series showed odds ratio greater than 2 (Table 3). In three populations, including USA [3]; French Canadian [19] and European [7] controls with >32 CAG were found. After independent exclusions of more precise risk estimates (Fig. 5C), such as Van Damme et al., 2011 (OR 2.70; 95% CI 2.20–3.29) [5]; Lee et al., 2011(OR 2.73; 95% CI 2.33–3.20) [3]; Elden et al., 2010 (OR 2.68; 95% CI 2.22–3.25) [2]; and Gellera et al., 2012 (OR 2.71; 95% CI 2.26–3.27) [20] the direction and magnitude of risk effect was no longer modified. Similar approach with exclusion of the most influential case-control series (the above data and Ross et al., 2011 [17], Van Langenhove et al., 2012 [16]) resulted in significantly heterogeneous risk estimates (combined OR = 2.34; 95% CI 1.94–2.83, χ^2^ = 40.04, df = 4, P = 0.0000) for CAG ≥32 repeats associated with ALS risk across populations. Overall our meta-analysis confirmed that the more accurate estimates in all populations are achieved when alleles with ≥30 CAG are used as cut-off for intermediates and the effect is more robust when CAG tract expansion is close to the upper limit of the *ATXN2*intermediate length. Moreover, it supported our findings on the pathological nature of *de novo* mutations found in the family reported here.

## Discussion

In SCA2, similarly to other triplet repeat disorders, the *de novo* mutations are thought to result from enlargement of the CAG repeat tract during father-to-child transmissions of pre-expanded alleles (23–30 CAG) to a pathogenic range of ≥34 CAG. Here we report three ALS cases coinciding with ≥30 CAG *ATXN2* alleles. For the first time, expansion of the normal repeat tract of ≤25 CAG, were shown to expand to a CAG repeat range causing or contributing to disease. *De novo* mutations in polyglutamine (polyQ)-related genes causing diseases have been reported in few SCA7 [21]–[23], one SCA6 [24] and a small number of Huntington’s Disease cases [25]. Regarding the *ATXN2* gene, there is no consistent evidence of novel mutations arising from non-pathogenic alleles (13–31 CAG). Schöls et al., [26] interpreted as *de novo* mutation a father-to-daugther transmission with 34 CAG (currently recognized as pathogenic) expansion to 41 CAG in daugther presenting as a sporadic SCA2 case. The father died from cancer at age of 65 years without exhibiting ataxia. Alonso et al., [27] reported on a Mexican sib-ship pair where the parents (both with 22/22 CAG) appeared to transmit an intermediate-length allele with 33 CAG to a child with early SCA2 onset. Unfortunately, in the previous studies, neither STR nor SNPs haplotypes were shown preventing a clear conclusion on *de novo* mutations. Van Damme et al. [5] suggested in a cases-control study a possible *de novo* origin for expansions of ≥32 CAG linked with sporadic ALS in one family, but no clear conclusion was made as DNA samples from siblings and parents were not available. In this study, we present three confirmed *de novo* mutations associated with ALS (transmissions T1, T3 and T4) in a single family. ALS in this family appeared first as sporadic but presented as familial following Mendelian pattern of inheritance. The familial form of ALS connected with intermediate *ATXN2* alleles has further been supported in a French-Canadian cohort showing a stronger association of alleles with more than 29 CAG in *ATXN2* with familial rather than sporadic ALS [19]. In our family, autosomal dominant inheritance pattern may be applied for the occurrence of ALS with *de novo ATXN2* expansions. The lacking of genetic heterogeneity with other prominent ALS genes (Table S1), the concomitant segregation of the intermediate and full CAG repeat expansion in our proband (III-13) and III-16 respectively (both individuals affected), and its absence in 80 chromosomes of the Cuban population satisfied the gold standard criteria pointing to a causative role. In two pedigrees, in which *SOD1*, *TARDBP*, *FUS*, and *ANG* mutations had been excluded, a co-segregation of the ALS phenotype with expanded *ATNX2* alleles has been reported [5]. The CAG expansions were either in the SCA2-parkinsonism or classic cerebellar SCA2, which, together with the reported genetic overlap [5] and without clinical heterogeneity or ALS phenotype modification [4], [5], [15], [19], [28] support our hypothesis of CAG expansion in *ATXN2* being monogenic ALS cause (at least for longer intermediate CAG expansions). In addition, *de novo ATXN2* CAG expansions may also contribute to apparent sporadic cases as reported by Elden et al [2]. This repeat behavior is supported by the meiotic and mitotic instability associated with large alleles [6]. All these observations may serve as a starting point for intergenerational risk estimations for CAG repeat instabilities in the large non-SCA2 expansions in *ATXN2* with ≥25 CAG.

Intermediate-length CAG expansions in *ATXN2* can lead to atypical SCA2 phenotype [8]–[10], however their association with ALS phenotype featured by shorter survival and onset at ∼54years of age helps to avoid misdiagnosis of ALS as SCA2 with motor neuron disease [29]. In our SCA2 population, ataxia combined with motor neuron phenotype has been noted [30], but such cases segregated within SCA2 pedigrees with cerebellar symptoms and a long disease duration excluding ALS. The intermediate *ATXN2* alleles associated either with familial or sporadic ALS arose as *de novo* CAG expansions unrelated to SCA2 families in our population.

Interestingly, one of the *de novo* mutations contributing to ALS crossed the pathogenic SCA2 threshold (35 CAG; T4 transmission) suggesting a similar mutagenic mechanism for both diseases (Fig. 6). The 35 CAG repeat expanded from a large normal allele which are overrepresented in Cuba [6] confirming their role as a source for new expansions.

The allele with 25 CAG repeat with only one CAA interruption in II-7 was transmitted as the 35 CAG expansion to III-13 (Fig. 4A,B). Similar allele would be the disease-causing mutation in II-2 and it is plausible to assume that in the preceding generation (grandparents and parents healthy), sequential loss of CAA in the 25 CAG allele resulted in CAG enlargements leading to ALS phenotype in successive generations (bottom model in Fig. 6).

Retrospective studies made in mortality archives between years 2001 and 2006 in Cuba showed low frequency of ALS, but Holguín province was third in the rating of death by ALS [33]. An introduction of a predisposed founder chromosome in this region would be proposed to contribute considerably to the ALS incidence in this region.

While the CAG sequence in SCA2 is pure, the ALS-related expansions are interrupted by CAA [4], [34] suggesting different origins. Two internal CAG repeat structure either with single or three CAA interruptions in sporadic ALS case series was interpreted as multiple mechanisms for *ATXN2* repeat expansions [17]. We observed a trend of pure CAG tracts gaining and removal of CAA interruptions in successive generations as shown in case II-10 (CAG 23+8) versus III-6 (CAG 8+8+4+8), respectively, indicating that both expansions share the same ancestor as previously proposed [35], and that mutational mechanism driving the removal or gaining CAA interruptions might occur concurrently.

Using microsatellites haplotype analysis, we found that the *de novo* mutations originate from the same ancestors although the extragenic microsatellites underwent some changes. On the other hand, we found SCA2 families in which the haplotype remained unchanged across generations [6], [36], [37]. This suggests that there may be regions in the vicinity of *ATXN2* which can interact genetically and epigenetically [38] and delineate and/or contribute further to ALS phenotype. This hypothesis is in accordance with the study Lahut et al. [15].

Two intragenic SNPs were used for haplotype analysis: rs695871 (G/C) and rs695872 (T/C) in the first exon of *ATXN2* gene situated 177 and 106 bp, respectively downstream from the CAG tract. The G→C substitution results in Val to Leu change while the T to C polymorphism is silent (Arg residue).The haplotype variants are CC or GT [17]. The CC haplotype was present in the 25 CAG allele, which expanded to intermediate CAG (Fig. 3B).This haplotype was confirmed also by the identification of the (CCG)_2_CCC sequence which is useful for haplotype discrimination [39] and evolutionarily conserved in primates [34]. The CC haplotype was connected with uninterrupted CAG tract and associated with intergenerational instability and ALS disease development in our three patients. This haplotype was reported in Indian population as enriched inSCA2 founders and in a subset of predisposed normal repeats, while the GT variant was found in the healthy population reducing the repeat sequence instabilities due to the presence of several CAA interruptions [34].Taken together, these data suggest that the presence of the CC haplotype, loss of CAA interruptions and the repeat size are prerequisite for CAG tract instability while the GT variant appears to stabilize the repeat even in large normal and intermediate CAG expansions and to prevent the SCA2-related mutagenesis. Surprisingly, however, the ALS cases carrying GT variant with three CAA interruptions developed the disease earlier than those with CC variant and less interruptions. Moreover, the ALS patients with three interruptions had shorter CAG than patients with fewer CAA interruptions [40].These observations suggest that besides the CAG repeat size, the discrete changes in *ATXN2* gene sequence probably affecting ataxin-2’s physiological function may be a risk factors for ALS development and ‘genetic hits’ facilitating the disease.

The effect of (CCG)_2_CCC (encoding poly-proline tail varying from 1–4) on CAG instability and possibly ALS phenotype should be also considered as it may contribute to both to the CAG (in)stability increasing the number of continuous triplets folding as hairpin, and to the content of prolines residues which lowers the complexity of unstructured sequence regions in the N-terminal tail of ataxin-2 [41].

Our meta-analysis further confirms the observation that the increased risk for ALS is specifically associated with long intermediate *ATXN2* repeats of ≥30 CAG and is greater with larger expansions. The heterogeneity observed in the combined risk is explained by longer intermediate alleles as the main factor. Pending other factors such as sample selection, gender ratio, SNPs haplotype or CAG calling approaches must be warranted and homogenized in further studies. Regarding this last technical point, the heterogeneity may reside in the range where authors don’t use sequencing methods, but for ≥27 CAG, none heterogeneity is expected, and great part is driven for the risk associated with CAG size variation only.

High number of intermediate alleles with 27–31 CAG not associated with any phenotype [6] complicates the interpretation of the “ALS threshold”. Only two out of 25 cases carrying intermediate CAG (32 and 33 CAG) had mild SCA2 phenotype and all intermediate alleles segregated within SCA2 pedigrees [6]. However, in a single family here, we showed three *de novo* mutated alleles penetrating with ALS disease. The question is what factors differentiate intermediate-length alleles segregating in SCA2 families from those associated with ALS? Our study point to a possible cis-acting factor, as STR haplotype changes were identified in all the *de novo* mutations.

In several back-to-back reports, *ATXN2* intermediate alleles associated with ALS have been defined differently across the populations, e.g. in the USA ≥27 CAG [2], in Europe ≥30 CAG [3], in Belgium and Netherland ≥32 CAG [5], suggesting a range of at-risk alleles with 27–30 CAG and alleles causing the disease with ≥31 CAG. This view is corroborated by recent studies showing escalating effect for alleles sized with 27Q, 29Q, 31Q and 32Q in the accumulation of phosphorylated and truncated TDP-43 in response to cell stress [42], [43]. Both post-translational modifications of TDP-43 are hallmark features of ALS pathology [44].

In conclusion, we identified de novo CAG expansions in *ATXN2* causing ALS. The mutational mechanism involved the loss of CAA anchors in large normal alleles on a predisposed genetic background, leading to a subsequent CAG instability. Intermediate ATXN2-ALS alleles segregated as *de novo* mutations in families in the general population, highlighting the necessity for providing *ATXN2* genetic testing in ALS patients.

## Methods

### Patient’s Resources and Clinical Characterization

First diagnosis of all patients was performed in the neuromuscular consultation at Clinical and Surgical Hospital Lucía Íñiquez Landín, Holguín, Cuba. Genetic status of the patients was not known at the time of first consultation. These individuals were enrolled in the research since they either met the revised El Escorial revisited criteria [45] for definite ALS or were related to ALS patients.

This study included a cohort of 17 individuals, four with familial ALS and 13 relatives. All studied subjects signed an informed consent form after being explained the purpose and methods of the study. All studies were approved by the review boards and ethics bureau of Center for Research and Rehabilitation of Hereditary in Holguin. Specifically, genealogical data were obtained for each family member and genetic DNA information was generated in regard to CAG expansions in the *ATXN2* gene aimed at determination of the founder allele.

The DNA testing of *ATXN2* was performed at the Department of Molecular Neurobiology of the National Center for the Research and Rehabilitation of the Hereditary Ataxias (CIRAH), Holguín. Since 1993, specialized database is available in this center enabling to confirm that there were no relationships between ALS cases and the 124 SCA2 families in the Cuban population. The genealogy database includes information from 124 multigenerational SCA2 families with more than 10.000 members across 15 generations, with names, demographic, genetic and clinical information.

### Determination of *ATXN2* CAG Repeat Size and SNIPs Haplotype

Peripheral blood leukocytes were extracted using EDTA as anticoagulant and genomic DNA was isolated using standard methods after informed consent was signed by patients or their guardians. In addition to SCA2, a complete set of polyQ diseases-related genes (SCA1, 3, 6, 7, 8, 17, HD) were analysed, with no remarkable findings. CAG repeat length in *ATXN2* was determined using Alfexpress II sequencing system and CAG fragments were separated with ReproGel™ high resolution system (GE Healthcare, Buckinghamshire, UK). PCR was performed as previously reported [6]. For detecting single nucleotide polymorphisms (SNPs), DNA was amplified with SCA2-FP3 and SCA2-RP3 primers [17] and using PCR conditions as previously reported in Ramos et al., 2010 [35], using 1 unit of TopTaq polymerase per reaction (Qiagen, Mainz Germany). For Short Tandem Repeat (STR) sequencing, oligonucleotides used for fragment analysis were also used for sequence reactions. Gel extraction was performed using GFX™ pCR DNA and Gel Band Purification kit (GE Healthcare, Buckinghamshire, UK) and the sequencing reactions were developed on Beckman Coulter CEQ8000 automated sequencing equipment with GenomeLab 10.2 software (Beckman Coulter, Inc. Belgium) at IPK. For sequencing, samples were blinded for the IPK investigators. Reactions were also done in both sense using FP3-RP3, and SCA2A primers, resulting contigs were assembled and compared with the Reference Sequence NG_011572.1. ALS patients were not genotyped for SOD1 and FUS mutations, but we exclude the contribution of the pathogenic GGGGCC expansions of C9ORF72 by using both repeat-primed PCR assays [31], [32].

### Short Tandem Repeat Haplotyping

Three chromosome 12 microsatellite CA repeat markers were used to establish the haplotypes in this study. Markers D12S1333 and D12S1332, with approximate 200 kb telomeric and 350 kb centromeric location, respectively from the gene, and the third marker, Dl2S1672, is located in the first intron of the *ATXN2* gene. PCR reaction was followed by Gene Scan analysis for STR allele identification. Reactions were performed in 13 µl containing ∼ 10 mM Tris-HCl (pH 8), 1.5 mM MgCl_2_, 50 mM KCl, 10% DMSO, 250 mM dNTPs, 100 ng of each of the primers, 1.5 U Taq-polymerase, and ∼100 ng genomic DNA. The following primer pairs and annealing temperatures were used: Dl2S1332a-b Cy5-GCC AGG TAC AGT GGC TC/CTG GGA CCA CAG GTG TAG at 60°C, D12S l333a-b Cy5-TTC AGG TGG TAC AGC CGT/CAT CAG AAG GCT TCA TAG GAA T at 50°C and D12Sl672a-b Cy5-CAG AGG GAG ATT CCA TCC AA/CGG TTT GAC AAG TTTCGA GA at 50°C. STR lengths were determined by Alfexpress II sequencing system and the PCR fragments were separated with ReproGel high resolution system. Internal (100 and 300 bp) and external (50–500-step, 50 bp) Alfexpress ladders were used to determine the fragment size. Traces were analyzed using the software Allelelink according to the manufacturer’s specifications.

### Meta-analysis

Meta-analysis was performed using odds ratio as measure of overall ALS risk, and the data were retrieved from the literature. Data were tabulated and processed in the program EPIDAT 3.1 of the Pan-American Health Organization (PAHO). We searched the literature investigating the *ATXN2* intermediate alleles as risk factors for ALS and performed a meta-analysis when applicable of case–control association (Table 3). Potentially useful studies were identified in review articles and through PubMed and Scopus, using the key words: ALS, ataxin-2 intermediate alleles and polyQ. The content of studies was also assessed manually to avoid redundant or unrelated data. We considered all studies published between 2010 until 2012. We avoided double counting using only the largest available published dataset from any study described in more than one published article. Given the heterogeneity in the nomenclature and thresholds of intermediate alleles we first considered threshold for intermediate alleles as originally authors reported in each work (i.e. 24, 27 and ≥30) (see also Table 3).

Thresholds for 24, 27, 30, 32 CAG repeats used here as starting point in our meta-analysis, were in accordance to the current literature reporting, where these thresholds were previously tested either as statistical significant or functional in each study (for details about rationale for thresholds see Text S1). For discriminating specific thresholds we applied component analysis, first analyzing the risks beginning with ≥24/27 CAG, ≥30 CAG, then we eliminated the contribution of the last alleles and the last cut-off was ≥32 CAG. The Dersimonian and Laird’s test was used to estimate the heterogeneity between relative risks in each metanalysis. When significant heterogeneity was absent, odds ratio and 95% confidence intervals (CIs) were calculated using a fixed-effects model while when significant heterogeneity was present, a random-effects model was used. Publication bias was investigated using funnels plots and both Begg’s and Egger’s tests. The significance level was set to P = 0.05.

We included 7, 471 ALS cases and 13,059 controls (ratio ∼1.75 control/cases) harboring 12 studies and including samples from North America, Europe, French-Canadian population, China, Italy, Belgium (Flanders), Germany and Turkey, published between 2010 and 2012 (Table 3). The studies were identified by the search strategy described above, met the inclusion criteria, and contributed to the meta-analysis (Table 3). Reports related with cases-controls studies in intermediate-length *ATXN2* CAG expansions and ALS in other languages than English were not found. There was no overlap in reporting ALS cases from the same country as authors did not specify whether samples were the same, which is not possible regarding that are different referral centers representing different regions. Lattante et al., 2012 [46] reported cases of sporadic ALS with *ATXN2* CAG expansions, but this study had no cases-controls design, therefore it was not included in our meta-analysis. Other two North-American studies [40], [47] were not included because were considered overlapping with Elden et al., 2010 [2]. During the revision of the article two additional studies has been published recently, two original from Italy and China, but were not included and did not modify our metanalysis [48], [49]. In the Chinese study, the repeat in *ATXN2* ranged between 30–35 CAG [48].

## Supporting Information

Table S1 Major Mutations in ATXN2-ALS case series.
(XLSX)

Text S1 Rational for polyQ thresholds used in Meta-Analysis.
(DOC)

Text S2 Abstract in Japanese.
(DOCX)
