# Supplementary material for: De Novo Mutations in Ataxin-2 Gene and ALS Risk
Source: PLoS One. 2013 Aug 6;8(8):e70560. doi: 10.1371/journal.pone.0070560 (PMC3735591; doi:10.1371/journal.pone.0070560)
Supplement: Text S1 — Rational for polyQ thresholds used in Meta-Analysis. (DOC) [file pone.0070560.s002.doc]

**Supplementary Text 1**

Why were the thresholds of 24, 27, 30 CAG repeats used in the meta-analysis?

The thresholds of 24, 27, 30, 32 CAG were used our meta-analysis as a starting point and, were in accordance with recent literature: i) The value of 24 CAG was firstly proposed by Elden et al. in 2010 [2]. The authors found that it was significantly associated with ALS in 915 ALS vs 980 neurologically normal controls in USA. To improve the stringency, they refined the starting point to ii) 27 CAG using ROC analysis. This threshold was validated by the group of Dr. Guy Rouleau in French-Canadian population [19] and confirmed in a Chinese and a Flemish study [8, 5]. In 2012, Laffita-Mesa et al. proposed that alleles with ≥27-31 CAG were somatically unstable, and using ROC curve were discriminated from those with 20-26 CAG but not from ≥32-34 CAG [6]. Moreover, Hart and Gitler found different behavior on apoptosis activation between alleles with 27, 28, and 29 compared to 32 CAG [42]. The threshold of 27 CAG was refined to iii) 30 CAG by Lee et al. in 2011 [3] with a stronger effect with larger CAG repeat size. This fact is in agreement with several reports related to late-onset SCA2 cases with 32-34 CAG [8,9,10]. Furthermore, Gispert et al. confirmed that the treshold of 30 CAG is valid in European populations [3,7]. Finally, Van Damme et al. , 2011 in a Belgian ALS cases-control study found genetic overlap of ALS and SCA2 with full pathogenic expansions (33, 34, 35, 39 CAG) and none clinical concordance [15]. This observation was not accepted in general [29]. Van Damme et al. found and proposed the threshold of 29 CAG [15]. Corrado et al. in 2011 and Lahut et al. in 2012 determined 32 CAG as the threshold in the Italian and Turkish population, respectively [4,15]. Importantly, Ross et al., 2011, found association of ALS and progressive supranuclear palsy with intermediate alleles of ≥32 CAG in a retrospective analysis of clinicopathologically confirmed [17].
