# Supplementary material for: De Novo Mutations in Ataxin-2 Gene and ALS Risk
Source: PLoS One. 2013 Aug 6;8(8):e70560. doi: 10.1371/journal.pone.0070560 (PMC3735591; doi:10.1371/journal.pone.0070560)
Supplement: Text S2 — Abstract in Japanese. (DOCX) [file pone.0070560.s003.docx]

**Abstract (Japanese)**

Ataxin-2遺伝子(ATXN2)の病的CAGリピート伸長は、脊髄小脳変性症2型(SCA2)の 遺伝的原因である。最近、同遺伝子は、パーキンソニズムと関連し、筋萎縮性側索硬化症(ALS)の遺伝性リスクを増加させることが報告された。我々は常染色体優先遺伝性ALSとATXN2のde novo変異の関係について報告する。 この知見は、

CAGリピート伸長に関連する神経変性の病理に含まれる新しい突然変異へのソースとして、長い正常ATXN2アリルの役割について、人口研究に基づいた我々の以前からの推測を支持する。de novo変異はメタアナリシス法によって証明された非ALS/SCA2 リスクアリルから伸長を起こした。 ALSのリスクはATXN2の中間伸長と同様に SCA2アリルとも関連していた。ALSの高いリスクとして病的CAGリピートが関連していることが、メタアナリシスによって明らかにされた。
